# Patient‐specific quality assurance for extremely small field sizes using 3D in vivo dosimetry in fractionated stereotactic radiosurgery for brain metastases

**DOI:** 10.1002/acm2.70417

**Published:** 2025-12-18

**Authors:** Kenichi Jumonji, Jun Takatsu, Naoya Hara, Ruiheng Fan, Risa Shimomura, Tatsuya Inoue, Kotaro Iijima, Noriyuki Okonogi, Naoya Murakami, Naoto Shikama

**Affiliations:** ^1^ Department of Radiation Oncology Juntendo University Graduate School of Medicine Bunkyo‐ku Tokyo Japan; ^2^ Department of Radiology Tokyo Rosai Hospital Ohta‐ku Tokyo Japan; ^3^ Department of Radiology Juntendo University Hospital Bunkyo‐ku Tokyo Japan; ^4^ Department of Radiology Juntendo University Urayasu Hospital Chiba Japan

**Keywords:** 3D in‐vivo dosimetry, error detection sensitivity, patient‐specific QA, PerFRACTION, small field

## Abstract

**Background:**

Fractionated stereotactic radiosurgery (fSRS) using LINAC‐based volumetric‐modulated arc therapy (VMAT) has become widely adopted and achieved favorable outcomes. However, this technique involves extremely small field sizes, making pre‐treatment quality assurance (QA) with high‐resolution detectors essential.

**Purpose:**

This study aimed to evaluate the error detection sensitivity and utility of EPID using 3D in vivo dosimetry based on dose‐volume histogram (DVH) analysis for brain fSRS pre‐treatment QA.

**Methods:**

VMAT plans were generated for spherical planning target volumes (PTVs) with diameters of 1–3 cm centered in SRS MapCHECK. A monitor unit (MU) output error was introduced in increments, and the leaf gap width was systematically opened and closed. Finally, multi‐leaf collimator (MLC) shift errors were applied. The error detection sensitivity was evaluated with these intentional errors. We compared this with the SRS MapCHECK 2D gamma analysis conventionally used in pre‐treatment QA. For in vivo dosimetry, 3D dose calculations were performed with PerFRACTION using cine‐mode electronic portal imaging device images and log files. DVH parameters such as D98%, D95%, D2%, and average doses of the PTV were compared between the base plan and the error plans. The 2D gamma analysis was performed using global gamma of 3%/2 mm, 3%/1 mm, 2%/2 mm, 2%/1 mm, and 1%/1 mm.

**Results:**

The 2D gamma analysis revealed no MU output errors for the 1 cm target across all criteria. The 2%/1 mm criterion effectively detected errors in leaf gap width and MLC shifts in some cases. Conversely, all error types were detected by 3D in vivo dosimetry using D95% of the target.

**Conclusion:**

In pre‐treatment QA for brain fSRS, 3D in vivo dosimetry based on DVH analysis demonstrated superior error detection sensitivity compared to conventional 2D gamma analysis. Our results suggest that 3D in vivo dosimetry is a useful tool for pre‐treatment QA in brain fSRS.

## 1 INTRODUCTION

Volumetric‐modulated arc therapy (VMAT) facilitates the irradiation of tumors while sparing normal tissue. Specifically, VMAT‐based intracranial fractionated stereotactic radiosurgery (fSRS) for brain metastases can deliver high doses to small targets, limiting the irradiation of normal brain tissue.^1^, ^2^ Moreover, single‐isocenter multi‐target (SIMT) VMAT enables reduced treatment times relative to the conformal‐arc technique or Gamma Knife intracranial fSRS.^3^, ^4^, ^5^ Previous research has shown outstanding clinical results using VMAT‐based intracranial fSRS.^6^, ^7^, ^8^ The SIMT VMAT technique involves the complex irradiation of small segments for very small tumors located off‐isocenter. Consequently, pre‐treatment quality assurance (QA) with measurements should be required.

Various devices have been used for pre‐treatment QA.^9^, ^10^, ^11^ While chamber measurements are the standard for absolute dosimetric evaluation, there has been the problem of underestimation due to volume averaging effects in measurements with extremely small fields, such as brain fSRS.^12^ Problems have also been reported with detector resolution in the semiconductor detectors capable of true composite measurements recommended by the American Association of Physicists in Medicine (AAPM) Task Group (TG)‐218.^13^ In this regard, devices that focus on stereotactic irradiation and have excellent detector resolution, such as the semiconductor detector SRS MapCHECK (Sun Nuclear Corp., Melbourne, FL) and the myQA SRS (IBA Dosimetry, Schwarzenbruck, Germany), have attracted attention in recent years.^14^, ^15^, ^16^, ^17^ While the ArcCHECK (Sun Nuclear Corp.) has a detector array with a spacing of 10 mm, the SRS MapCHECK has a spacing of 2.47 mm, and the myQA SRS has a spacing of 0.4 mm, making them suitable for small field measurements. However, since the sensitivity area is 77 × 77 mm^2^ for the SRS MapCHECK, multiple measurements would be required in cases where the target is far from the isocenter. The myQA SRS has a larger sensitivity area of 140 × 120 mm^2^, but still requires multiple measurements with detector plane adjustments to ensure optimal measurement geometry for multiple targets.

The measured doses are generally compared with the calculated doses with gamma analysis.^13^, ^18^ However, it has been reported that there is little correlation between the gamma passing rates for a homogeneous phantom and the dosimetric errors from patient anatomy.^19^, ^20^, ^21^, ^22^, ^23^ This is especially true in brain fSRS, for which target volumes could be quite small. In such cases, even small dosimetric errors should be important. Previous studies have described a technique called measurement‐guided dose reconstruction (MGDR). This reconstructs three‐dimensional (3D) in vivo dosimetry using the dose distribution measured in a phantom.^24^, ^25^ MGDR allows QA for 3D in vivo dosimetry to be conducted in accordance with clinical protocols through visual assessment of dose distribution in a patient's anatomy and the use of dose–volume histogram (DVH) parameters. Nonetheless, MGDR is only feasible with commercial devices possessing detector resolutions of approximately 10 mm, such as the ArcCHECK and the Delta4 Phantom+ (ScandiDos, Uppsala, Sweden). Its application to brain fSRS is challenging due to the limitation in the calculation resolution. A method for performing 3D in vivo dosimetry has been reported that uses log files generated by a linear accelerator during irradiation.^26^, ^27^, ^28^ This log file QA technique allows 3D in vivo dosimetry at the same resolution as the dose computations in the treatment planning system (TPS). However, issues have been found with log files being unable to correctly acquire the multi‐leaf collimator (MLC) positions during irradiation.^29^, ^30^

Recent studies have investigated 3D in vivo dosimetry that combines electronic portal imaging device (EPID) measurements and log files, which is within the capabilities of the SunCHECK platforms (Sun Nuclear Corp.).^31^, ^32^ The available software executes 3D in vivo dosimetry by capturing the MLC positions during irradiation using EPID images obtained in the cine mode. This overcomes any uncertainty regarding the MLC position data in the log files, allowing 3D in vivo dosimetry at the same calculation resolution as the TPS. There have been limited reports on the utility of the SunCHECK platform for 3D in vivo dosimetry with small fields. Therefore, this study aimed to evaluate the error detection sensitivity and usefulness of 3D in vivo dosimetry using the SunCHECK platform as the QA device for brain fSRS compared with conventional 2D gamma analysis methods for small fields.

## 2 METHODS

### 2.1 Error detection sensitivity as pre‐treatment quality assurance in fractionated brain stereotactic radiosurgery

To evaluate the error detection sensitivity of brain fSRS as a pre‐treatment QA tool for each QA method, plans with various intentional errors were generated. First, SRS MapCHECK was inserted into StereoPHAN (Sun Nuclear Corp.), and computed tomography (CT) images were scanned. CT scanning was performed using the Aquilion LB (Canon Medical Systems, Otawara, Tochigi, Japan) at 120 kVp, with a slice thickness of 2 mm. The CT images were then imported into RayStation, v. 10A (RaySearch Laboratories, Stockholm, Sweden). The physical density of the CT images was replaced with polymethyl methacrylate (PMMA) (mass density = 1.19 g/cm3). Spherical dummy planning target volumes (PTVs) with diameters of 1, 2, and 3 cm were generated in the center of the SRS MapCHECK.

A single full‐arc VMAT plan was generated for each dummy PTV using a 6 MV flattening filter‐free (FFF) beam, and the plan was normalized to cover 95% of the volume of each PTV with a prescription dose set at 5 Gy per fraction. The Collapsed Cone Convolution algorithm was used for dose calculation, which was performed with a 1 mm grid size. The field size was fixed at a jaw size of 3 × 3 cm^2^, and the field was generated using the MLC.

The plans for each dummy PTV were defined as the base plans. Three types of intentional errors were systematically introduced to assess the error detection sensitivity of each QA method. These were (i) monitor unit (MU) output errors, (ii) MLC open/close errors, and (iii) MLC misalignment errors. The MU output error was produced by changing the MU of the base plan by 1% within a range of −2%–+2%. This simulation was designed to account for differences between the output generated by TPS at each control point and the actual irradiation during treatment. The MLC open/close error was produced by using the script function of RayStation to systematically open and close the leaf gap width at each control point by −2, −1, −0.5, +0.5, +1, and +2 mm. The MLC shift error was simulated by using the script function of RayStation to systematically shift the MLC position at each control point +1 and +2 mm toward the B side from the base plan. The base plan and the intentional error plan were irradiated using TrueBeam (Varian Medical Systems, Palo Alto, CA).

### 2.2 Measurement devices

#### 2.2.1 Conventional 2D gamma analysis

SRS MapCHECK was used for the 2D gamma analysis. SRS MapCHECK is designed to measure irradiation from any angle by implementing corrections for angle dependence.^15^ A previous study classified SRS MapCHECK as a high‐resolution detector and found that it was able to detect MLC position errors.^17^ Therefore, SRS MapCHECK was selected to perform the conventional 2D gamma analysis in this study.

Dose calculation was performed using a 6X‐FFF beam with 100 MU for a 5 × 5 cm^2^ field.^33^ The criteria for 2D gamma analysis were global 3%/2 mm, 3%/1 mm, 2%/2 mm, 2%/1 mm, and 1%/1 mm. The threshold was set at 10% for all criteria. The calculated dose of the base plan by RayStation was compared with the measured doses of the base plan and intentional error plans. Analysis was performed using SNC Patient software (Sun Nuclear Corp.).

#### 2.2.2 3D in vivo dosimetry

The SunCHECK platform was used to evaluate the error detection sensitivity of 3D in vivo dosimetry as a pre‐treatment QA tool in brain fSRS. The SunCHECK platform is capable of fast dose calculation with a graphical processing unit using a different beam model from that of the TPS.^32^ The dose calculation algorithm uses collapsed cone convolution/superposition (C/S). DoseCHECK is a module of the SunCHECK platform that performs secondary dose calculation using plans, CT, and structure information exported from the TPS. Another module is PerFRACTION, which provides a pre‐treatment QA tool. In this study, 3D in vivo dosimetry was performed using PerFRACTION's Fraction 0 option.^31^ Log files were used to acquire output, and the gantry and collimator angles at each control point, and the MLC positions were acquired from the cine EPID images using a novel algorithm. The platform also allows 3D dose calculations that account for delivery errors when irradiated with TrueBeam.

The dosimetric evaluation using 3D in vivo dosimetry was based on a comparison of the DoseCHECK and PerFRACTION DVH parameters. To evaluate the error sensitivity of the pre‐treatment QA tool, reference doses were calculated using DoseCHECK rather than RayStation to avoid the influence of the different calculation algorithms. The DVH parameters evaluated were D98%, D95%, D2%, and the mean dose of the dummy PTV. The error detection sensitivity of 3D in vivo dosimetry was evaluated based on the differences between DoseCHECK and PerFRACTION in the changes of the DVH parameters. The differences between these DVH parameters were calculated using the following formula.

$$Difference=\frac{\left(PF_{error}-DC_{base}\right)\;}{DC_{base}}\;\;\times 100\%$$

where DCbase and PFerror are the DVH parameters of the dose calculation results of DoseCHECK without errors and PerFRACTION with intentional errors, respectively.

## 3 RESULTS

### 3.1 Error detection sensitivity of conventional 2D gamma analysis with a high‐resolution detector

**FIGURE 1:**
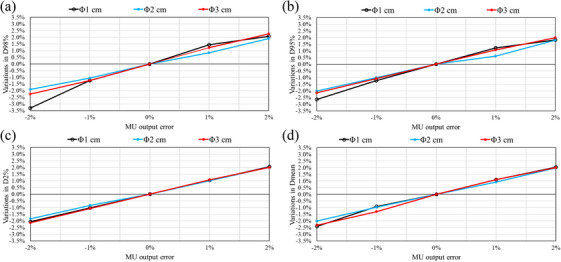
The gamma passing rates of various intentional errors found using SRS MapCHECK for fractionated brain stereotactic radiosurgery. Three types of intentional errors were introduced (a–c) MU output errors; (d–f) MLC open/close errors; (g–i) MLC shift errors. (a), (d), and (g) show the results with a 1 cm target diameter. (b), (e), and (h) show the results with a 2 cm target diameter. (c), (f), and (i) show the results with a 3 cm target diameter. All gamma analyses threshold values were set to 10%. MLC, multi‐leaf collimator; MU, monitor unit.

Figure 1 shows the results of the gamma analysis for three types of errors (MU output errors, MLC open/close errors, and MLC shift errors) for three different target sizes. Error detection sensitivity was assessed using the amount of variation in the gamma passing rate for the intentional errors. It can be seen that the solid line in the figure with a distance‐to‐agreement (DTA) of 1 mm has a steeper change in the gamma passing rate than the dotted line with a DTA of 2 mm. This trend was consistent for all intentional error types. The highest detection sensitivities were seen with 2%/ 1 mm and 1%/ 1 mm. When the target size was small (diameter of 1 cm), even a slight error in the MLC open/close error caused a sharp decrease in the gamma passing rate. As the target size increased (to 2 and 3 cm), the changes in the gamma passing rate for the same error tended to be more moderate. The gamma passing rate also changed asymmetrically according to whether the error was positive (in the MLC opening direction, the power increased) or negative, as shown in Figure 1a–f. The gamma passing rate tended to either remain unchanged or improve when the error was positive and then decrease rapidly, but the amount of decrease differed depending on the error direction. As shown in Figure 1g–i, the variations in gamma passing rate for intentional MLC shift errors were dependent on the target size. When the target size was small, MLC position errors could not be detected by the gamma passing rate.

### 3.2 Error detection sensitivity of 3D in vivo dosimetry

**FIGURE 2:**
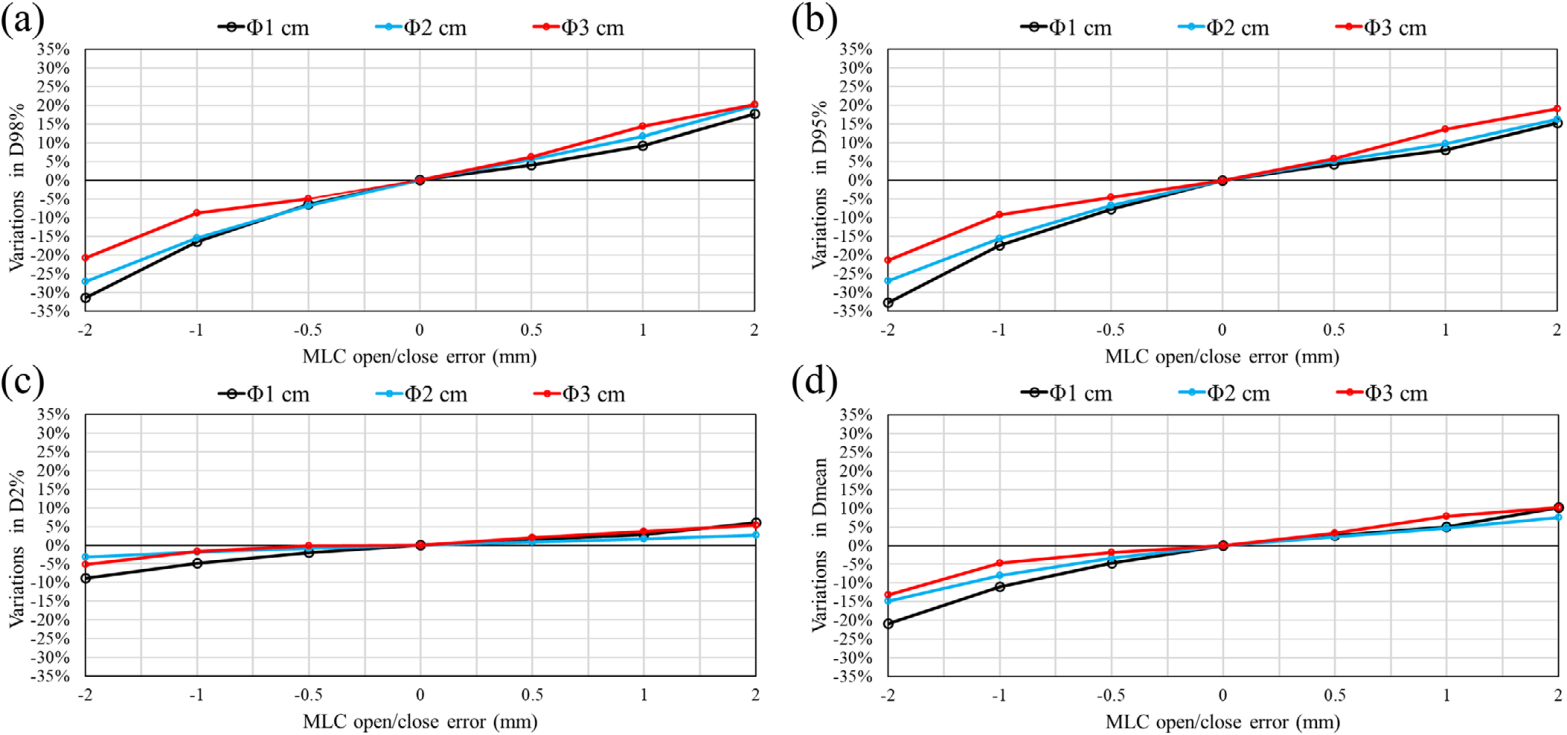
Variations in DVH parameters using 3D in vivo dosimetry with intentional MU output errors for fractionated brain stereotactic radiosurgery. DoseCHECK and PerFRACTION measurement differences from the base plan are shown for (a) D98%, (b) D95%, (c) D2%, and (d) Dmean. MU, monitor unit.

Figure 2 shows the results of 3D in vivo dosimetry using the DVH parameters (D98%, D95%, D2%, and Dmean) of DoseCHECK and PerFRACTION over MU output errors ranging from −2% to +2%. For all target sizes, 3D in vivo dosimetry showed a clear linear relationship between the DVH parameters and the output error magnitude. Sensitivity was consistent across all DVH parameters and target sizes.

**FIGURE 3:**
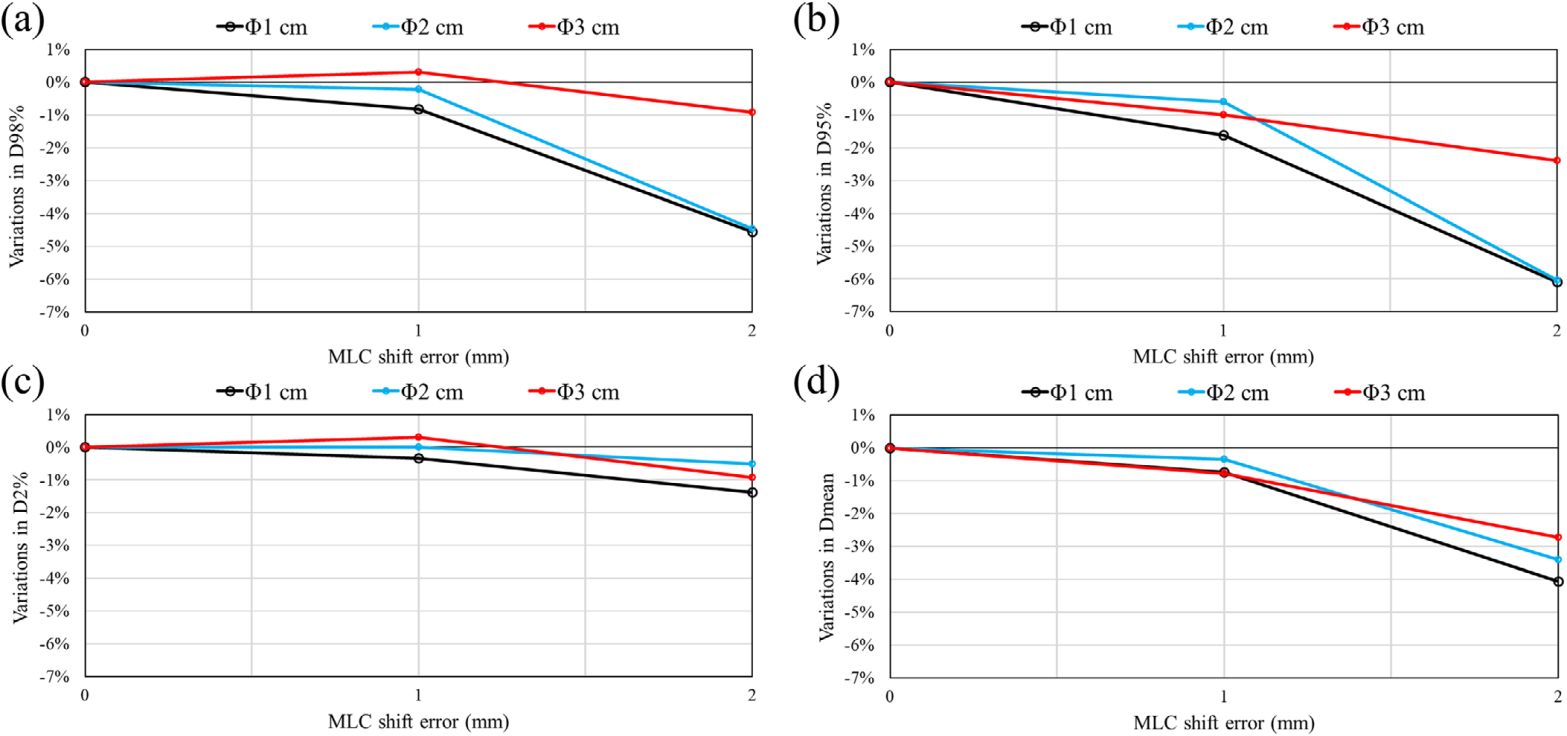
Variations in DVH parameters using 3D in vivo dosimetry with intentional MLC open/close errors for fractionated brain stereotactic radiosurgery. DoseCHECK and PerFRACTION measurement differences from the base plan are shown for (a) D98%, (b) D95%, (c) D2%, and (d) Dmean. MLC, multi‐leaf collimator.

Figure 3 shows the results of 3D in vivo dosimetry for MLC open/close errors ranging from −2 to +2 mm. Compared to MU output errors, MLC position errors resulted in greater changes in the DVH parameters as the target size became smaller. D98% and D95% showed similar trends, indicating excellent error detection sensitivity.

**FIGURE 4:**
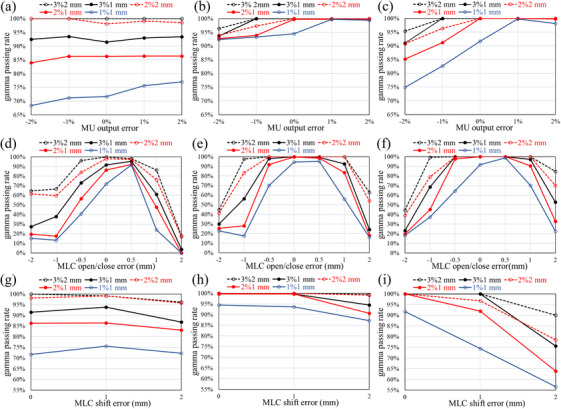
Variations in DVH parameters using 3D in vivo dosimetry with intentional MLC shift errors for fractionated brain stereotactic radiosurgery. DoseCHECK and PerFRACTION measurement differences from the base plan are shown for (a) D98%, (b) D95%, (c) D2%, and (d) Dmean. MLC, multi‐leaf collimator.

Figure 4 shows the results of 3D in vivo dosimetry for MLC shift errors ranging from 0 to +2 mm. Although the variation was smaller than that seen with the MLC open/close errors, all parameters clearly detected the shift errors. D95% and Dmean in particular showed excellent error detection sensitivity.

In contrast to the 2D gamma analysis results (Figure 1), which showed asymmetric and irregular gamma passing rate behavior for different error types, the 3D in vivo dosimetry results showed consistent linear relationships for all error types.

### 3.3 Comparison of treatment planning system calculations and measured doses with intentional errors

**FIGURE 5:**
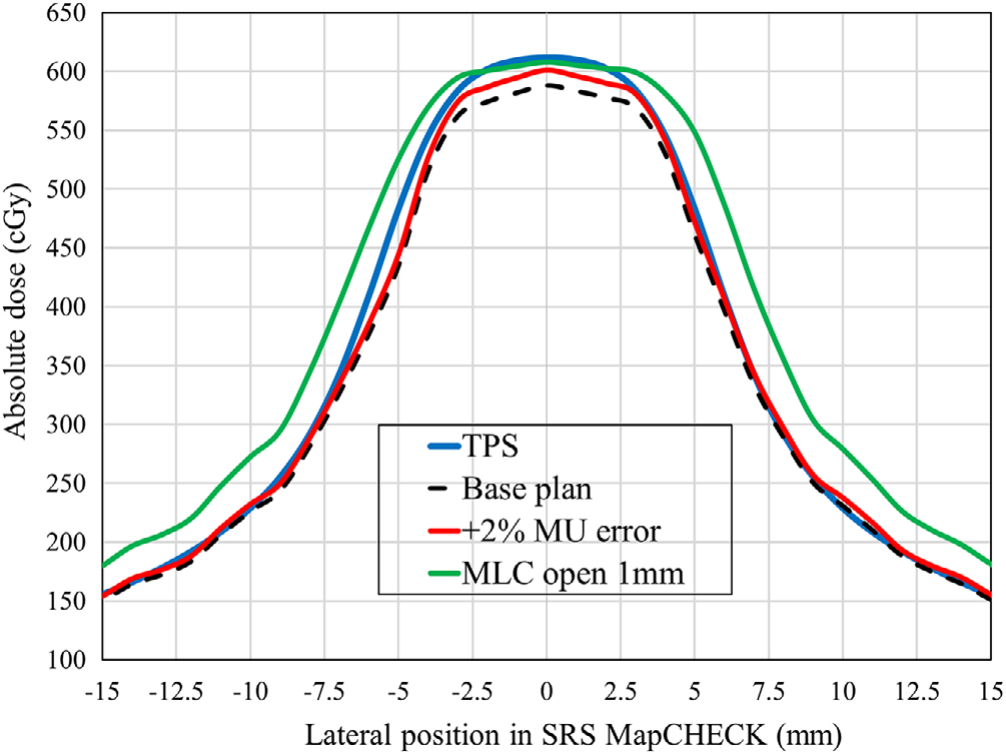
Comparison of the lateral dose profiles for fractionated brain stereotactic radiosurgery with a 1 cm diameter target between TPS calculations, base plan measurements, +2% MU errors, and MLC open errors of 1 mm. MLC, multi‐leaf collimator; MU, monitor unit; TPS, treatment planning system.

Figure 5 compares the lateral dose profiles of a 1 cm diameter target for TPS calculations, base plan measurements, +2% MU errors, and MLC open errors of 1 mm. As shown in Figure 5, the TPS calculated dose was approximately 4% higher than the measured dose of the base plan. Therefore, when an intentional MU error was applied to increase the output, the measured dose became closer to the TPS calculation, and the gamma passing rate improved. A similar effect was observed with MLC opening errors, which could not be correctly detected by conventional 2D gamma analysis due to the calculation uncertainty of TPS beam modeling for extremely small field sizes.

## 4 DISCUSSION

This study compared the error detection sensitivity and utility of DVH‐based 3D in vivo dosimetry using the SunCHECK platform as a QA tool for VMAT‐based brain fSRS with those of the conventional 2D gamma analysis method.

Two‐dimensional gamma analysis of SRS MapCHECK found that MU output errors resulted in minor variations in the gamma passing rate for the target with a diameter of 1 cm. This indicates that none of the criteria were able to detect the errors (see Figure 1a). As can be seen in Figures 1b,c, 2%/1 mm and 1%/1 mm provided superior detection sensitivity for MU output errors with 2 and 3 cm diameter targets. Dunn et al. have previously recommended 2%/1 mm as the error detection sensitivity for pre‐treatment QA in brain fSRS using SRS MapCHECK.^34^ However, AAPM Medical Physics Practice Guideline 9.b recommends a gamma analysis criterion of 3%/1 mm for pre‐treatment QA.^35^ For MU output errors, the error detection sensitivity was insufficient. In contrast, 3D in vivo dosimetry was able to detect MU output errors from all DVH parameters regardless of the target size, as shown in Figure 2.

Numerous studies have reported on the detection sensitivity of MLC position errors using high‐resolution detectors.^34^, ^36^, ^37^ These have consistently demonstrated that MLC position errors can be detected at 2%/1 mm using high‐resolution detectors. As shown in Figure 1d–f, 2%/1 mm and 1%/1 mm criteria were more sensitive to MLC open/close errors than more relaxed criteria, such as 3%/2 mm. The 1%/1 mm criterion was particularly effective with the smallest target (diameters of 1 cm). This showed gamma passing rates < 20% for MLC errors of ± 1 mm, which dropped to nearly 0% for errors of ± 2 mm. These results align with those of a previous study, recommending stricter gamma criteria.^11^ Generally, as the target size increases, the relative impact of dosimetric errors from intentional errors becomes smaller. Our study results showed a similar trend. However, our study also revealed that the sensitivity of 2D gamma analysis to small MLC errors (± 0.5 mm) decreases as the target size increases from 1 to 3 cm. This reduction in error detection sensitivity for larger targets could allow clinically meaningful errors to be overlooked when using conventional 2D gamma analysis. Additionally, the improvements in gamma passing rates due to underlying discrepancies between the TPS calculations and the measurements were observed for positive MU errors and MLC opening errors, as illustrated in Figure 5. Dose calibration for SRS MapCHECK was performed using the manufacturer‐recommended 5×5 cm^2^ field size, following the report by Xu et al.^33^ They reported better QA results for brain fSRS with small targets using this calibration. Therefore, the uncertainty in the SRS MapCHECK measurements in this study was minimized. The dosimetric error between the TPS‐calculated values and the base plan measurements shown in Figure 5 was considered to be due to the uncertainty in the TPS beam modeling. This highlights a fundamental limitation of 2D gamma analysis for small‐field dosimetry in brain fSRS. Thus, this finding suggests that 2D gamma analysis was inadequate for detecting intentional errors. Small field size beam modeling remains a major challenge in current clinical practice. Falco et al. reported greater variability in small field size beam modeling between institutions.^38^ Wu et al. recommended extending the output factor registered in the TPS to 1×1 cm^2^ to ensure accurate dose calculation precision in small fields.^39^ In contrast, the 3D in vivo dosimetry approach demonstrated consistent error detection capabilities regardless of target size. As shown in Figure 3, variations in DVH parameter occurred in response to MLC open/close errors across all target sizes, with D98% and D95% exhibiting the highest sensitivity.

As shown in Figure 1g–i, analysis of MLC shift errors revealed clear differences between the results obtained by conventional 2D gamma analysis and 3D in vivo dosimetry. Error detection sensitivity increased with larger target sizes. With a 1 cm target, sensitivity to MLC shift errors was insufficient for both 2%/1 mm and 1%/1 mm criteria. These results contrast with those reported by Dunn et al., who used SRS MapCHECK with 2%/1 mm and 1%/1 mm gamma criteria to successfully detect systematic MLC shift errors of 0.5 and 1 mm.^34^ However, this discrepancy can be explained by methodological differences. Dunn et al. evaluated 21 targets ranging from 0.4 to 8.1 cm^3^, whereas this study focused on a single, 0.5 cm^3^ spherical target (diameter of 1 cm). The complexity of the MLC field differs between plans with multiple targets and the single target. Therefore, the impact of systematic shift errors on dose distribution is greater. We demonstrate that, unlike 2D gamma analysis, 3D in vivo dosimetry consistently and reliably detects MLC shift errors for all target sizes, as shown in Figure 4. This approach successfully identifies dose differences due to MLC shift errors, regardless of target size, and shows the highest sensitivity with D95% among the evaluated DVH parameters.

In this study, the movement of the leaf traversing the target was suppressed because strict constraints were not applied to the maximum dose in the target during optimization. Consequently, the effects of the tongue‐and‐groove effect on the measurement results were minimized. In clinical practice, the collimator is typically rotated to suppress the tongue‐and‐groove effect; however, in this study, the collimator angle was set to 0 degrees. Therefore, the impact of this collimator angle setting on the conclusions of this study was considered limited. This study had several limitations that should be addressed. First, this study focused on spherical single targets. This was a simplification of real clinical brain fSRS cases, which often involve multiple irregularly‐shaped targets. While this approach enabled us to systematically evaluate error detection sensitivity across different target sizes, its applicability to multi‐target treatments with more complicated MLC fields and dose distributions requires further research. Additionally, this study limited its analysis to systematic errors (uniform MU changes and MLC errors), whereas random errors and combinations of different error types can occur in clinical practice. Future studies should also consider verification using stricter 2%/1 mm criteria in 2D gamma analysis, although this study could not determine acceptable levels for such criteria. Further research is needed to establish appropriate 2D gamma analysis criteria for clinical practice. However, despite these limitations, our findings strongly suggest that 3D in vivo dosimetry provides superior error detection for brain fSRS compared to conventional 2D gamma analysis, especially for small targets. Another important consideration is variability in TPS modeling accuracy across institutions. As Figure 5 shows, discrepancies between TPS calculations and measurements can substantially affect QA results, especially for small fields with substantial modeling discrepancies. The magnitude and characteristics of these discrepancies can vary depending on the TPS commissioning process, beam modeling techniques, and measurement equipment used at a given institution. Also, note that when obtaining the MU during irradiation from log files, there may be a systematic error in the calibration. This error can causean uncertainty in the detection of the irradiation output error by in vivo dosimetry. Therefore, multi‐center validation studies are needed to confirm the generalizability of our findings and develop standardized protocols for 3D in vivo dosimetry in pre‐treatment QA of brain fSRS.

## 5 CONCLUSION

We compared the error detection sensitivity of DVH‐based 3D in vivo dosimetry using the SunCHECK platform with that of conventional 2D gamma analysis in brain fSRS. Even with strict criteria (2%/1 mm and 1%/1 mm), no output errors or MLC position errors were detected for a target with a diameter of 1 cm. However, when appropriate DVH parameters such as D95% were used, 3D in vivo dosimetry demonstrated higher error detection sensitivity in small field sizes than conventional 2D gamma analysis for MU output errors and MLC position errors. Hence, this study demonstrates the utility of 3D in vivo dosimetry in pre‐treatment QA for brain fSRS, which involves irradiation of very small volumes.

## AUTHOR CONTRIBUTIONS

Kenichi Jumonji collected and analyzed the data. Kenichi Jumonji and Jun Takatsu prepared the manuscript. Jun Takatsu is the corresponding author. Naoya Hara, Ruiheng Fan, and Risa Shimomura collected the data. All co‐authors revised the manuscript. Dr. Murakami and Dr. Shikama provided supervision of the manuscript. Dr. Shikama provided final approval of the manuscript.

## CONFLICT OF INTEREST STATEMENT

This study was supported by a research grant from Toyo Medic Corporation, Tokyo, Japan.
